# Patient perceptions, opinions and satisfaction of telehealth with remote blood pressure monitoring postpartum

**DOI:** 10.1186/s12884-021-03632-9

**Published:** 2021-02-19

**Authors:** Nicole A. Thomas, Anna Drewry, Susan Racine Passmore, Nadia Assad, Kara K. Hoppe

**Affiliations:** 1 University of Wisconsin-Madison, School of Nursing, Signe Skott Cooper Hall, 701 Highland Avenue, Madison, WI 53705 USA; 2 Department of Obstetrics and Gynecology, School of Medicine and Public Health, University of Wisconsin, Madison, WI USA; 3 University of Wisconsin-Madison, Collaborative Center for Health Equity, School of Medicine and Public Health, Madison, WI USA; 4 University of Wisconsin-Madison Survey Center, Madison, WI USA

**Keywords:** Postpartum hypertension, Remote patient monitoring, Telehealth, Participant satisfaction, Qualitative evaluation

## Abstract

**Background:**

Our aim was to conduct a post participation survey of respondent experiences with in-home remote patient monitoring via telehealth for blood pressure monitoring of women with postpartum hypertension. We hypothesized that the in-home remote patient monitoring application will be implemented with strong fidelity and have positive patient acceptability.

**Methods:**

This analysis was a planned secondary analysis of a non-randomized controlled trial of telehealth with remote blood pressure patient monitoring for postpartum hypertension compared to standard outpatient monitoring in women with a hypertension-related diagnosis during pregnancy. In collaboration with survey experts, we developed a 41-item web-based survey to assess 1) perception of quality of care received, 2) ease of use/ease to learn the telehealth program, 3) effective orientation of equipment, 4) level of perceived security/privacy utilizing telehealth and 5) problems encountered. The survey included multiple question formats including Likert scale responses, dichotomous Yes/No responses, and free text. We performed a descriptive analysis on all responses and then performed regression analysis on a subset of questions most relevant to the domains of interest. The qualitative data collected through open ended responses was analyzed to determine relevant categories. Intervention participants who completed the study received the survey at the 6-week study endpoint.

**Results:**

Sixty six percent of respondents completed the survey. The majority of women found the technology fit easily into their lifestyle. Privacy concerns were minimal and factors that influenced this included age, BMI, marital status, and readmissions. 95% of women preferred remote care for postpartum follow-up, in which hypertensive type, medication use and ethnicity were found to be significant factors in influencing location of follow-up. Most women were satisfied with the devices, but rates varied by hypertensive type, infant discharge rates and BMI.

**Conclusions:**

Postpartum women perceived the telehealth remote intervention was a safe, easy to use method that represented an acceptable burden of care and an overall satisfying method for postpartum blood pressure monitoring.

**Trial registration:**

ClinicalTrials.gov identification number: NCT03111095 Date of registration: April 12, 2017.

## Background

Hypertensive disorders are one of the most common complications of pregnancy in the United States. Approximately 10% of pregnancies are affected nationwide [1–3]. While guidelines for antepartum and intrapartum management of hypertension are numerous, recommendations for monitoring for hypertension in the postpartum period have just started to emerge over the past decades despite the fact that hypertensive disorders are one of the leading reasons for postpartum readmission, morbidity and mortality [3].

Blood pressure (BP) decreases within 48 h following delivery and increases 3–6 days postpartum [1, 4, 5]. The American College of Obstetrics and Gynecology (ACOG) suggests that obstetric providers monitor the BPs of women with gestational hypertension, preeclampsia or superimposed preeclampsia inpatient, or that equivalent outpatient surveillance be performed for the immediate 72 h postpartum and again at 7–10 days postpartum or earlier in women with symptoms [1]. Of note, 50 to 70% of women do not follow up postpartum [6–8]. To address this gap we developed a telehealth with remote monitoring intervention devised for daily home BP monitoring. All data was transmitted to clinical providers on a daily basis [6]. We conducted a single-site non-randomized controlled trial of telehealth with remote monitoring and linked interventions for management of postpartum hypertension. The intervention was associated with reduced hospital readmissions compared to standard care (1 [0.5%] vs. 8 [3.7%], adjusted relative risk 0.12; 95% confidence interval (CI): 0.01–0.96) [9]. After providing informed consent, postpartum women prior to hospital discharge were assigned to and dispensed a remote monitoring unit that securely transmitted individual data to a central monitoring platform via Bluetooth technology leveraged by Honeywell (now Resideo) Lifestream Clinical Monitoring solution. To increase equitable utilization of services among intervention participants, all necessary equipment was provided and telehealth services was capable via wi-fi, internet, or cellular data. Equipment dispensed to each intervention participant included a Genesis Touch tablet, automatic blood pressure cuff, scale and pulse oximeter. Prior to hospital discharge the participants were trained on use of equipment and were requested to submit biometric data daily. Registered nurses trained in the research protocol assessed participant data daily and used nurse-driven BP algorithms for initiation, titration and cessation of anti-hypertensive medication as indicated [6].

ACOG defines telehealth as “technology-enhanced health care framework that includes services such as virtual visits, remote patient monitoring, and mobile health care.” [7] This technology has been used for blood pressure monitoring in the non-obstetrical population [8] as well as broader applications managing heart failure, anticoagulation and chronic pulmonary disorders [10]. Survey results regarding remote monitoring in non-obstetrical patients indicate a high rate of acceptance due to a sense of empowerment and lack of disruption in daily routine [10]. This technology has been shown to be feasible in obstetrical patients’ [11–13] and initial studies regarding telehealth blood pressure monitoring have demonstrated high patient satisfaction both antenatally [14] and in the postpartum period [15, 16]. Furthermore, a recent study indicated a low rate of privacy concerns with telehealth monitoring of postpartum blood pressures [17].

The objective of this study is to assess patient perspectives and experiences regarding daily postpartum blood pressure monitoring via telehealth with remote patient monitoring. Of great contribution to this study is the self-administered questionnaire (SAQ). It is more detailed than previous studies which have evaluated patient’s perspectives and experiences regarding remote monitoring for postpartum hypertension. Additionally, at the time of our study there was no validated survey to evaluate areas of interest related to remote monitoring. This survey was designed to ascertain overall opinions and to also specifically query, burden of care, ease of use and satisfaction separately. Furthermore, this is the first study to our knowledge which will analyze patient opinion by hypertension type, healthcare utilization, medication use and infant/maternal factors. In turn, this SAQ has the potential to guide future survey studies for telehealth with remote patient monitoring for blood pressure monitoring of women with postpartum hypertension. Last, the questionnaire could also be useful for investigators in other specialties who are studying remote patient monitoring for blood pressure surveillance, if adapted to reflect their study.

Our hypothesis was that intervention participants would find the remote blood pressure monitoring equipment to be easy to use, a secure way to submit health data, not overly burdensome and an overall satisfying way to receive postpartum care of their hypertension.

## Methods

We conducted a cross-sectional, post-participation web-based survey study to assess 1) perception of the quality of care received, 2) ease of use/ease to learn the telehealth program, 3) effective orientation of the equipment, 4) level of perceived security/privacy utilizing telehealth and 5) problems encountered using the health equipment devices.

Inclusion criteria for the parent intervention study and subsequently this survey were women admitted for delivery of their neonate with any of the following hypertensive diagnoses: chronic hypertension, gestational hypertension, preeclampsia or eclampsia. At six-weeks postpartum the study equipment was returned and an online self-administered questionnaire (SAQ) was distributed to each intervention participant who had completed the study. The SAQ was sent to the email address they had identified at time of enrollment through Qualtrics Survey Service (Qualtrics, Provo, UT). Participants received a package of diapers at enrollment as well as a $15 gift card at the conclusion of the intervention study period at 6 weeks postpartum. Participants provided written informed consent. The study was approved by the University of Wisconsin Institutional Review Board (IRB # 017–003 approved 03/21/2017).

All methods were performed in accordance with the relevant guidelines and regulations (Declaration of Helsinki).

### Survey development

The survey was developed in in a collaborative effort between the experts at the University of Wisconsin Survey Center and our team of women’s health physicians. It included 41questions which assessed: 1) perception of the quality of care received, 2) ease of use/ease to learn the telehealth program, 3) effective orientation of the equipment, 4) level of perceived security/privacy utilizing telehealth and 5) problems encountered using the health equipment devices. The survey included 4-point Likert-type scale items (1=” Not at all” or “Never” to 4= “A great deal” or “Extremely” or “Extremely often”). Additional items pertained to demographics; time required for vital sign submission (1–15 min); dichotomous Yes/No responses; and open-ended questions. The open-ended questions encouraged written responses on individuals’ personal experience of equipment (BP cuff, scale, monitor), and suggestions for improvement. Questions included 1) Please tell us more about the problems you have had regarding the tablet, 2) BP cuff, 3) scale, and 4) Please tell us anything you can think of that would make using the health devices a better experience for you.

Types of hypertension were divided into chronic hypertension, gestational hypertension, preeclampsia without severe features and all other preeclampsia which included preeclampsia with severe features, HELLP and eclampsia. Healthcare utilization was assessed by Emergency Room (ER) visit or readmission, length of initial hospital stays and length of initial postpartum stay. Maternal demographics were collected including insurance type, age, marital status, and race and medication use, specifically medications prior to delivery, medications at discharge and increase or initiation of medications after discharge. Finally, infant parameters included NICU admission, gestational age at delivery and discharge of mother and infant together.

Statistical analysis of the quantitative survey responses included both descriptive statistics and two forms of logistic regression. All quantitative survey questions were analyzed to assess the breadth of results. Logistic regression was performed on a subset of questions specifically addressing the domains 1) ease of use, 2) burden of care, 3) satisfaction and 4) privacy. We wanted to determine if any relationships existed between these specific four domains and types of hypertension, health care utilization and demographic characteristics of the respondents.

These were divided into types of hypertension, healthcare utilization, maternal demographics, medications, and infant parameters. We used binary logistic regression for dependent variables that were dichotomous, and an ordered logistic regression model for scale type variables. The odds ratio (OR) and associated standard error (SE) and associated *p*-value were reported for each independent variable to assess which independent variables were significantly associated with the dependent outcome of each question of interest. When dealing with scale variables such as the ones we have, research shows that it is more appropriate to use an ordered logistic model rather than OLS regression by either failing to demonstrate significance of certain variables or underestimate the effect of variables in the model [18]. All analyses were performed with Stata version 15.0 (College Station, Texas).

Text responses were coded using an iterative content analysis approach in which some common categories were identified across items [19]. NVivo 12 software was used to facilitate qualitative data management and analysis for the open-ended items.

## Results

**Fig. 1:**
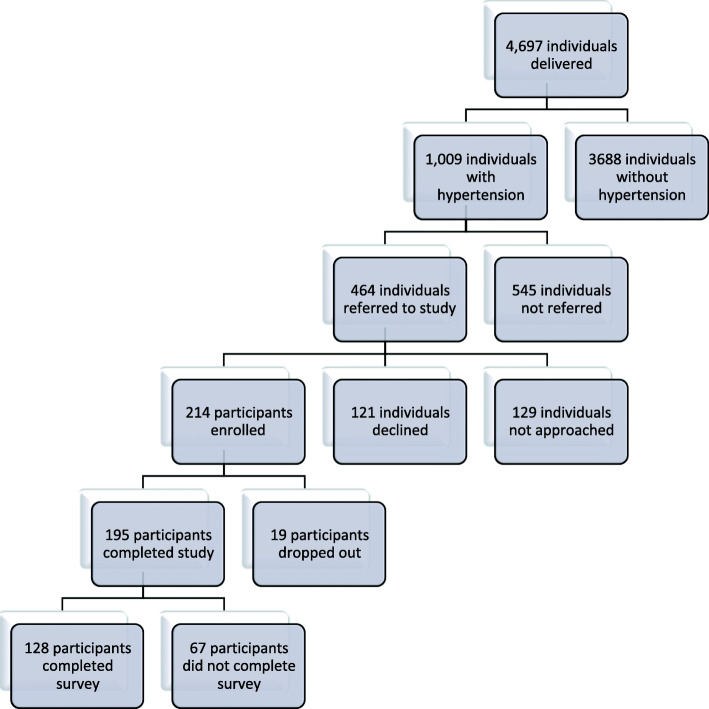
Trial Enrollment and Intervention Participation

All intervention participants were included in the final analysis of the primary study using to intent to treat principals, therefore the comparative study group to the survey group includes all 214 participants (see Fig. 1 for trial enrollment and intervention participation). Survey respondent characteristics are compared to the overall study intervention participants in Table 1. The characteristics of the survey respondents were similar to the overall study intervention participants with the exception that survey respondents were significantly more likely to be married (113, 88% vs 163, 76%; *p* = 0.006). The survey results for all questions are detailed in Table 2. Survey results of the univariate logistic regression analysis are described in Table 3.

**Table 1:** Characteristics of survey respondents compared to overall study intervention participants (*N* = 128)

Characteristics	Survey Respondents (*N* = 128)	Intervention Participants (***N*** = 214)	*p*-value
**Age (years)**	32 ± 4.6	31 ± 5.0	0.06
**BMI at delivery (kg/m**^**2**^**)**	37 (31.0–41.1)	35 (24.4–36.0)	0.588
**Non-Hispanic white**	112 (88)	175 (85)	0.537
**Marital status (married)**	113 (88)	163 (76)	0.006
**Hypertension diagnosis**			0.786
Chronic without Superimposed Preeclampsia)	10 (8.0)	21 (9.8)	
Chronic with Superimposed Preeclampsia	10 (8.0)	22 (10.3)	
Gestational	42 (33.0)	62 (29.0)	
Preeclampsia	76 (59.3)	131 (61.2)	
Severe features	40 (31.0)	51 (23.8)	
Without severe features	35 (27.0)	80 (37.4)	
HELLP	4 (3.0)	11 (5.6)	
Eclampsia	1 (0.8)	1 (0.4)	
**Insurance status, n (%)**			0.055
Private	118 (92)	185 (86)	
Medicaid	9 (0.7)	30 (14)	
Other	1 (0.007)	–	

Continuous data presented as mean ± SD for parametric and median (interquartile range) for nonparametric distributions. Categorical data presented as N (%)

Statistical tests including Wilcoxon rank sum test, Chi-square or fishers exact test were used where appropriate

*BMI* Body Mass Index, *HELLP* Hemolysis, elevated liver enzymes, low platelets

**Table 2:** Results to all qualitative questions

Survey Question	Total responses	Mean	Standard Deviation	Number responding Yes (n,%)
**Ease of Use**				
How complicated are mHealth’s instructions	**127**	**0.25**	**0.67**	
How much mental effort does mHealth require	**127**	**0.59**	**0.74**	
How easily does mHealth fit into your lifestyle	**127**	**2.65**	**0.96**	
How easy was it to get help^a^	**43**	**3.33**	**0.87**	
How confident do you feel using the mHealth devices	**127**	**3.46**	**0.79**	
Genesis Touch Monitor				
• how hard is it to use	**126**	**0.16**	**0.49**	
• how organized is it	**127**	**3.27**	**0.78**	
• how helpful are its prompts	**127**	**2.57**	**1.34**	
How easy is the blood pressure cuff to use	**127**	**3.44**	**0.79**	
How hard is the weight scale to use	**126**	**0.37**	**1.06**	
Did you ever get help for your problems with the devices^a^	**67**			**24 (35.82)**
How reasonable is the amount of time it takes to record?				
• your blood pressure	**127**	**3.46**	**0.77**	
• your weight	**127**	**3.47**	**0.79**	
How burdensome is it to record				
• your blood pressure daily	**127**	**0.85**	**0.93**	
• your weight daily	**127**	**0.78**	**0.98**	
How long in minutes did it take to measure and record:				
• your blood pressure				
o1 min				**118 (90.77)**
o5 min				**11 (8.46)**
• your weight				
o1 min				**122 (94.57)**
o5 min				**5 (3.88)**
Have you had any problems using the Genesis Touch monitor	**127**			**49 (38.58)**
Have you had any problems using the blood pressure cuff	**127**			**108 (85.04)**
Have you had any problems using the scale	**127**			**107 (84.25)**
**Privacy**				
How secure do you feel submitting your vitals	**127**	**3.41**	**0.76**	
Did you have concerns sending your vitals to your health care provider	**127**			**4 (3.15)**
Do you have enough control over your data	**127**			**113 (89.76)**
How much more:				
• in control of your own health do you feel	**128**	**2.91**	**0.9**	
• aware of your own health do you feel	**128**	**3.30**	**0.74**	
**Burden of Care**				
Did you have to go to an emergency room after discharge?	**128**			**14 (10.94)**
Did you have a hospital readmission?	**128**			**3**
• Was it a different hospital than your delivery hospital?^a^	**3**			**1**
Do you prefer going to clinic/hospital instead of using mHealth for postpartum follow-up	**128**	**0.31**	**0.78**	
How much would you recommend mHealth to others in your situation?	**127**	**3.49**	**0.79**	
**Satisfaction**				
How enjoyable are the mHealth devices to use	**127**	**2.41**	**1.06**	
How satisfied are you with the mHealth devices	**127**	**3.32**	**0.83**	
To what extent does using the mHealth tech make you feel safer?	**128**	**2.86**	**0.96**	
How often do you feel unsafe while using mHealth?^a^	**8**	**0.38**	**1.06**	
How fun is answering questions using the Genesis Touch Monitor	**127**	**2.24**	**1.03**	
Did the Genesis Touch Monitor have all functions you expected	**127**	**3.02**	**0.98**	
Do you like the touch screen technology on the Genesis Touch monitor	**127**	**3.43**	**0.74**	
Does using mHealth make you worry more, less or the same	**127**	**1.23**	**0.63**	
Survey Questions	**Total responses**			
**Qualitative Open-Ended Questions: Problems**				
Please tell us more about the problems you have had with the:				
• Monitor	***			
• BP Cuff	***			
• Scale	***			
Please tell us about anything that you can think of that would make using the health devices a better experience for you?	***			

Response values are in 4-point Likert-type scale items (ex. 1=” Not at all” or “Never” to 4 = “A great deal” or “Extremely” or “Extremely often”), *** qualitative results are summarized in the results section, survey questions using branch logic (^a^) attribute to low response rates

**Table 3:** Univariate logistic regression results

Domain	Question	Variable	Odds Ratio (OR)	Standard Error (SE)	95% CI
**Ease of use**	How complicated were the instructions of mHealth technology?	Maternal BMI	0.87*	0.05	0.78–0.97
		Marital status	0.08**	0.08	0.01–0.53
	How much mental effort is required to interact with the mHealth technology?	Mother’s age	1.1*	0.05	1.0–1.2
	How easily did using the mHealth technology fit in with your lifestyle?	Maternal BMI	0.95^+^	0.02	0.90–1.0
		ER visit/readmission	2.6^+^	1.4	0.87–7.7
	How easy was it to get help?	No significant variables			
	How confident do you feel using the mHealth devices?	ER visit/readmission	6.5*	5.3	1.3–32
**Privacy**	How secure do you feel submitting your vitals using the Genesis Touch monitor?	No significant variables			
	Do you have enough control over your data?	No significant variables			
**Burden of care**	To what extent do you prefer going to the hospital or clinic instead of using the mHealth technology at home?	No significant variables			
	How much would you recommend the mHealth technology to other women in your situation?	Gestational hypertension	12	17.4	0.71–204
		Preeclampsia without severe features	36*	54.7	1.86–701
		All other preeclampsia	24*	31	1.9–294
		Starting medication after discharge	4.1*	2.9	1.0–16.4
		Non-Hispanic White	7.6^+^	7.1	1.2–47.4
**Satisfaction**	How enjoyable are the mHealth devices to use?	All other preeclampsia	12.7*	13	1.7–94.2
		Chronic hypertension	8.8^+^	11	0.77–101
	Overall how satisfied are you with the mHealth devices?	All other preeclampsia	19*	26	1.3–283
		Maternal BMI	0.94*	0.03	0.88–0.99
		Infant discharging with mother	4.4^+^	3.48	0.90–21
		Starting medication after discharge	3.1^+^	1.9	0.94–10

*p < 0.05, **p < 0.01, ***p < 0.001, +*p* < 0.1

With respect to the ease of use domain, only 1.6% (2/128) of women felt the instructions for use were very or extremely difficult, 0.9% (1/128) of women felt the technology required an extreme amount of mental effort, 59% (75/127) reported the technology easily (very or extremely) fit in their lifestyle, 80% (34/43) felt help was readily accessible when needed, and 80% (101/127) of women felt confident using the devices. Burden of Care was minimal, with only 4.7% (6/128) of women preferring to go to the hospital or clinic instead of using technology at home, and 91% (115/127) of women responding that they would recommend this care to women in the same situation. Overall, 84% (107/127) reported that they were very or extremely satisfied with the equipment. Privacy concerns were minimal as well; 88% (112/127) of women felt secure transmitting protected health information, and 90% (114/127) felt they had sufficient control over their data.

Upon performing logistic regressions (Table 2) significant relationships were found between patient characteristics and three of the four domains – ease of use, burden of care and satisfaction. There were no significant differences between groups regarding privacy metrics.

Examining **ease of use,** data by question and associated variables demonstrated the perception of how complicated the instructions were influenced by maternal BMI (OR, 0.87; SE, 0.05; 95% CI, 0.78–0.97) and marital status (OR, 0.08; SE, 0.08; 95% CI, 0.01–0.53). The perception of amount of mental effort required to use the equipment was associated with mother’s age (OR, 1.10; SE, 0.05; 95% CI, 1.0–1.2) and how easily the technology fit with the patient’s lifestyle was influenced by maternal BMI (OR, 0.95; SE, 0.02; 95% CI, 0.90–1.0) and emergency room (ER) visit or readmission (OR, 2.6; SE, 1.40; 95% CI, 0.87–7.7). ER visit or readmission also influenced respondents” confidence level with the devices (OR, 6.48; SE, 5.31; 95% CI, 1.3–32).

In terms of **burden of care**, women were more likely to recommend this technology to other women if they had gestational hypertension (OR,12.0; SE,17.4; 95% CI, 0.71–204), preeclampsia without severe features (OR, 36.0; SE, 54.7; 95% CI, 1.86–701), all other preeclampsia (OR, 24; SE, 31; 95% CI, 1.9–294), started medications after discharge (OR, 4.1; SE, 2.9; 95% CI 1.0–16.4) or were non-Hispanic white (OR, 7.6; SE, 7.1; 95% CI, 1.2–47.4). Additionally, open-ended responses revealed that respondents found the intervention to yield an acceptable level of burden of care. For example, respondents noted that::

*[The program] eased the burden of having to not go out in the snow for appointments with my newborn!! -Patient 1*

And*,*

*“I personally really enjoyed using this product, I didn't want to be in the hospital any more than I had to … I am very satisfied and happy to have gotten the chance to take this device home, it made me feel safer.” – Patient 2*

Lastly, for **satisfaction** with remote blood pressure monitoring the analysis showed that women with other forms of preeclampsia found the devices more enjoyable to use (OR, 12.7; SE, 13.0; 95% CI, 1.7–94.2) as did women with chronic hypertension (OR, 8.8; SE, 11.0; 95% CI, 0.77–101). Overall satisfaction rates were higher with women with all other forms of preeclampsia (OR, 19; SE, 26; 95% CI, 1.3–283, mothers who were discharged home with their infants (4.4, 3.5) and who started on medications after discharge (OR, 3.1; SE, 1.9; 95% CI, 0.94–10). The higher the maternal BMI the lower the overall level of satisfaction reported with the devices (OR, 0.94; SE, 0.03; 95% CI, 0.88–0.99). Narrative comments from open-ended questions also imply overall satisfaction. Examples include,

*“I don't think I would have been as well cared for with traditional office visit Medicare. My pressures were especially high just after discharge...which happened to be over weekend. I was distracted caring for my new baby and wouldn't have called the doctor on the weekend and certainly wouldn't have gone into a clinic or the emergency department with my new baby. So, my preeclampsia would have gone untreated without this technology. Thank you for this program! Even while pregnant as my pressures rose and my edema worsened, I felt my providers were dismissive of my concerns about a possible preeclampsia diagnosis...in retrospect, I am so disappointed in my obgyn office for not making this diagnosis during pregnancy and feel sure that they would have neglected to monitor and treat this disease postpartum as well. It is only because of this program that I received such excellent monitoring and care.*

*Again, thank you!” – Patient 3*

And,

*“I felt empowered, informed and safe. The nurses at telehealth made me feel like I mattered and were very informative. They even asked me questions about my recovery and my baby which made it much more personal. This program saved me a lot of stress and grief. I would recommend this to all women in my situation.” – Patient 4*

And,

“*The nurses and staff were all phenomenal in staying in touch if any concern arose. I felt so much better having these devices after pregnancy to monitor my vitals and get them under control.*” *– Patient 5*

Regarding suggestions for improvements, several respondents noted problems associated with the design of equipment (ex: scale too heavy to move, blood pressure cuff to small/large).

However, they most frequently noted problems with Bluetooth syncing and cellular data connectivity. These problems resulted in an inability for the Bluetooth enabled data collection devices to reliably sync the patient’s clinical data to the tablet for transmission to clinicians. In these instances, respondents submitted data through text messaging, or phone calls which accomplished the need to submit data but at the expense of privacy, increased effort and time.

For example, *“Sometimes the BP cuff wouldn't register with the device and I'd either have to repeat the test or text the information to the study coordinator. It gave me a lot of trouble at first. We had to unpair and then pair the machines. After that, it still happened, but much less often.” – Patient 6*

And,

*“It wasn't difficult, but it was an extra step and a little less privacy having to send my weight in texts.” – Patient 7*

Some respondents noted poor cellular data connectivity issues which at times resulted in the inability to transmit the synced data to the clinician. In these instances, when able, the respondent’s home internet service was connected, and cellular data turned off.

Finally, respondent comments stress the importance of having an intervention compatible with the busy and ever shifting lifestyle that comes with a new baby. One particular annoyance was the scheduling of data collection (BP, pulse & weight) at 9 am.

*The monitor would yell at me “Good morning, it is time to take your vitals” if I didn’t do it right at 9am. It would continue to do this every two minutes until I did take my vitals. This was frustrating for a new mom as some of the times I was feeding my newborn and other times I had just gotten to go back to sleep after a crazy night. There was no way to silence this alarm or turn the volume down on it. I had to call a clinician to have her change my alarm settings for it to stop yelling at me...it would have been nice to have more control over this part myself.*

*– Patient 8*

In the same way, mothers wanted more flexibility in system navigation. For example, *“Also, sitting through the blood oxygen monitoring when I didn't have a device to take the measurement was a waste of time--I was forced to sit through the tablet go through the motions of the oximeter instead of skipping ahead to the next step.” – Patient 9*

Again, this point was specifically important for new mothers.

*“It would be nice if you had a "submit these vitals?" option at the end that you had to accept so that if something was off or incorrect, you could redo that portion. My toddler stepped on the scale while I was trying to weigh myself a few times and it submitted both her weight alone once and my weight combined with her weight a second time. It would have been nice if I could have cancelled those weights and redone them without having to go back through the whole touch screen system after they were incorrectly submitted.” – Patient 10*

Finally, a few respondents noted that it would be helpful to have access to a history of their data submissions to self-monitor over the course of the intervention.

## Discussion

### Main findings

The results of this study confirm our hypothesis that remote blood pressure monitoring was generally accepted by postpartum women with a hypertensive disorder as an easy to use, secure system that was overall satisfying and did not represent an undue burden of care. These findings are also congruent with earlier studies [17, 20, 21] in this area which indicate high rates of patient satisfaction and recommendation of remote monitoring programs for future participants’ [15].

Our findings are the first to delineate specific groups who may be more amenable to this intervention. Younger respondents found less mental energy levels was required to use the devices, which may be expected as younger individuals may be more adept at using technology. The differences in ease of use metrics, in regards to marital status may also be due to variations in patient ages between married and unmarried groups although that analysis was not performed in this study due to the sample size limitations. Furthermore, respondents who had a postpartum ER visit or readmission felt most confident in using remote blood pressure technology. Our results suggest, women with conditions making them at increased risk of complications will feel secure and have broad adoption with engagement in remote monitoring.

This study also demonstrated that most respondents feel secure using remote blood pressure monitoring and that privacy and security concerns did not appear to be influenced by type of hypertension (listed in Table 1), healthcare utilization, maternal demographics, medications and infant parameters are not associated with perceptions of privacy or security concerns. In contrast, there were certain groups of women who were more likely to recommend remote blood pressure monitoring to other women in similar situations. Specifically, women with de novo blood pressure concerns related to pregnancy were more appreciative of the technology. One possible explanation for this trend is that unlike women with chronic hypertension, these individuals may be less familiar with home blood pressure monitoring and may not already have the necessary equipment.

Women who started medications after discharge also were more likely to recommend the program and be more satisfied with remote blood pressure monitoring. As initiation of a new medication can be a significant event for a woman, participants who start medications while in the telehealth program may be more apt to see the benefits of such a program.

Finally, similar to findings from recent studies [22, 23], while this program represented a more intense surveillance regimen than traditional office blood pressure checks, it had high satisfaction rates from respondents with chronic and pregnancy related hypertension, indicating potential broad acceptability among women. Women discharged with their infants also were more likely to be satisfied with remote monitoring, possibly because it eliminates the need to balance office blood pressure visits with the demands of newborn cares, additional children, and work obligations [23].

The most common suggestions from respondents to improve future iterations of the program include strengthening internet/Bluetooth connectivity support such as portable hotspot devices. Notably, this may also have the added effect of minimizing some privacy concerns; and through additional capability for customization (ex: data collection reminders; personal data history, etc.).

While there are certain groups that may find more benefit or satisfaction from remote blood pressure monitoring than others, these results indicate that most women will find value in this system regardless of demographic characteristics, hypertension type and medication use. This supports that from a patient perspective, remote blood pressure technology is likely to be acceptable to women when offered as an alternative to traditional hospital/clinic-based blood pressure surveillance for any individual who requires additional postpartum blood pressure surveillance as identified by ACOG.

### Limitations

Limitations of this study include the fact that it performed at a single site with a relatively homogenous population and small sample size. Furthermore, although the results indicate the respondents preferred telehealth to standard outpatient blood pressure monitoring, the study did not include a control group in which satisfaction rates and burden of care metrics were also assessed. Additionally, the lack of a control group does introduce the risk of volunteer bias into the results. Specifically, individuals who participate in a remote blood pressure monitoring program may have fewer privacy concerns that the general population and be more adept at technology. We acknowledge that the associated baseline variables used in our analysis were exploratory and we did not adjust for multiple comparisons so some findings of significance may be due to chance. Last, although the Genesis Touch Monitor came equipped with language settings other than English, due to financial restraints, a limitation of the study is that it was limited to English speaking participants. However, upon completion of the intervention study, we transitioned to a no-cost remote blood pressure monitoring program for postpartum hypertension in which all women affected by a hypertensive disorder of pregnancy are in invited to participate free of charge.

## Conclusions

Future directions include conducting a larger multicenter study with more patient diversity to validate the findings of this study. In the interim, this study indicates that telehealth with remote blood pressure monitoring has high rates of acceptance among respondents who view it as a secure way to submit protected health information and reduce the burden of care associated with postpartum blood pressure monitoring. As technology continues to advance, telehealth monitoring may represent an important method of data collection regarding postpartum blood pressure trends, improve compliance with blood pressure recommendations, and reduce readmission rates to ultimately improve maternal health.

## Data Availability

Data sets used and analyzed during the current study are available from the corresponding author on reasonable request*.*

## Abbreviations

BMI: Body Mass Index
BP: Blood Pressure
ER: Emergency Room
HELLP: Hemolysis, elevated liver enzymes, low platelets
OR: Odds Ratio
SE: Standard Error
